# Mesenchymal subtype neuroblastomas are addicted to TGF-βR2/HMGCR-driven protein geranylgeranylation

**DOI:** 10.1038/s41598-020-67310-0

**Published:** 2020-07-01

**Authors:** Michael E. Stokes, Jonnell Candice Small, Alessandro Vasciaveo, Kenichi Shimada, Tal Hirschhorn, Andrea Califano, Brent R. Stockwell

**Affiliations:** 1 Department of Biological Sciences, Columbia University, New York City, NY 10027 USA; 2 Present Address: Department of Medicine, Harvard Medical School, Chemical Biology and Therapeutic Sciences Program, Broad Institute, Boston, MA 02115 USA; 3 Department of Systems Biology, Columbia University, New York City, NY 10027 USA; 4 Present Address: Laboratory of Systems Pharmacology, Harvard Medical School, Boston, MA 02115 USA; 5 Department of Chemistry, Columbia University, New York City, NY 10027 USA

**Keywords:** Chemical libraries, Lipids, Mechanism of action, Small molecules

## Abstract

The identification of targeted agents with high therapeutic index is a major challenge for cancer drug discovery. We found that screening chemical libraries across neuroblastoma (NBL) tumor subtypes for selectively-lethal compounds revealed metabolic dependencies that defined each subtype. Bioactive compounds were screened across cell models of mesenchymal (MESN) and MYCN-amplified (MYCNA) NBL subtypes, which revealed the mevalonate and folate biosynthetic pathways as MESN-selective dependencies. Treatment with lovastatin, a mevalonate biosynthesis inhibitor, selectively inhibited protein prenylation and induced apoptosis in MESN cells, while having little effect in MYCNA lines. Statin sensitivity was driven by HMGCR expression, the rate-limiting enzyme for cholesterol synthesis, which correlated with statin sensitivity across NBL cell lines, thus providing a drug sensitivity biomarker. Comparing expression profiles from sensitive and resistant cell lines revealed a TGFBR2 signaling axis that regulates *HMGCR*, defining an actionable addiction in that leads to MESN-subtype-dependent apoptotic cell death.

## Introduction

Neuroblastoma is an extra-cranial pediatric tumor, responsible for approximately 15% of all pediatric cancer deaths^1^. Subtype classification and staging of disease has important prognostic implications for neuroblastoma (NBL) patients^2^. Approximately 25% of NBL tumors harbor amplifications of the *MYCN* locus (MYCNA), which correlates with high-risk disease and poor prognosis^3^. While significant progress has been made toward understanding the drivers of the MYCNA subtype, which includes the majority of MYCN amplification (MYCNA) events^4^, less is known about the regulatory and metabolic underpinnings of remaining NBL subtypes. Recent molecular characterization of high-risk primary tumors from the NCI TARGET Consortium and the European NRC database identified a novel molecular tumor subtype (MESN) characterized by a mesenchymal-like gene expression signature^4^, which strongly overlaps with that of mesenchymal glioblastoma (GBM)^5^. Further analysis indicated that 15–25% of NBL primary tumors are comprised in this aggressive MESN subtype^4^, suggesting that the identification of pharmacologically accessible dependencies within this subtype may provide an opportunity to improve treatment options for this patient population.

Network analysis of MESN NBL primary tumors revealed a distinct set of regulatory drivers that underpin this aggressive tumor phenotype. These “Master Regulator” (MR) proteins are transcription factors that act coordinately to establish and maintain MESN pathophysiology^4^. Subtype-specific activation of MR proteins may result in metabolic and signaling dependencies unique to the MESN subtype, thus providing researchers with suitable “Achilles’ heels” that can be targeted therapeutically. We thus hypothesized that the unique tumor architecture of MESN NBL may give rise to targetable metabolic dependencies that can be identified using appropriate cell models. We screened bioactive molecules across a panel of cell lines identified as high-fidelity models for both MESN and MYCNA NBL subtypes, based on conservation of MR proteins and regulatory network^4^. This screen revealed both the mevalonate and folate pathways as essential metabolic processes that support MESN subtype viability. Chemical inhibitors and genetic tools were then used to probe these pathways and uncover mechanisms through which they induce MESN-subtype-specific cell death.

Statins are commonly used cholesterol lowering medication that inhibit 3-hydroxy-3-methylglutaryl-CoA reductase (HMGCR), the rate-limiting step of cholesterol biosynthesis^6^. Six statin drugs have been approved for use in children and are well-tolerated in patients^7^. Subtype-specific statin sensitivity has been observed in a number of cancer cell line models^8–15^, but the mechanisms that underpin selectivity appear context dependent and have not been well defined in pediatric tumors. Here, we explored the mechanisms through which statins selectively induce cell death in MESN NBL, and reveal mechanistic relationships between the mevalonate pathway and regulatory drivers that define the MESN subtype.

### Targeted screen identifies MESN-selective metabolic inhibitors

To identify MESN-selective lethal compounds, we screened a collection of bioactive molecules across high-fidelity cell line models of the MESN and MYCNA NBL subtypes, leading to a set of molecules displaying elevated differential potency in MESN cell lines. To determine which cell models were most appropriate to represent the two subtypes, protein activity profiles from 39 NBL cell lines, generated using the VIPER algorithm^16^, were evaluated for enrichment of the MESN and MYCNA MR-protein activity signature generated from VIPER analysis of patient-derived gene expression profiles in two NBL cohorts^4^. For each cell line, we plotted its normalized enrichment scores (NES) (Supplemental Figure S1A)—representing the enrichment of the cell line’s differentially active proteins in MR proteins of the MYCNA and MESN subtypes, respectively. Based on this analysis, SK-N-AS and NLF were chosen as optimal, high-fidelity models of the MESN tumor subtype, while two common MYCNA cell lines, SK-N-Be2 and IMR-32, were counter-screened as optimal MYCNA subtype representatives to assess subtype-specific differential compound sensitivity.

**Figure 1:**
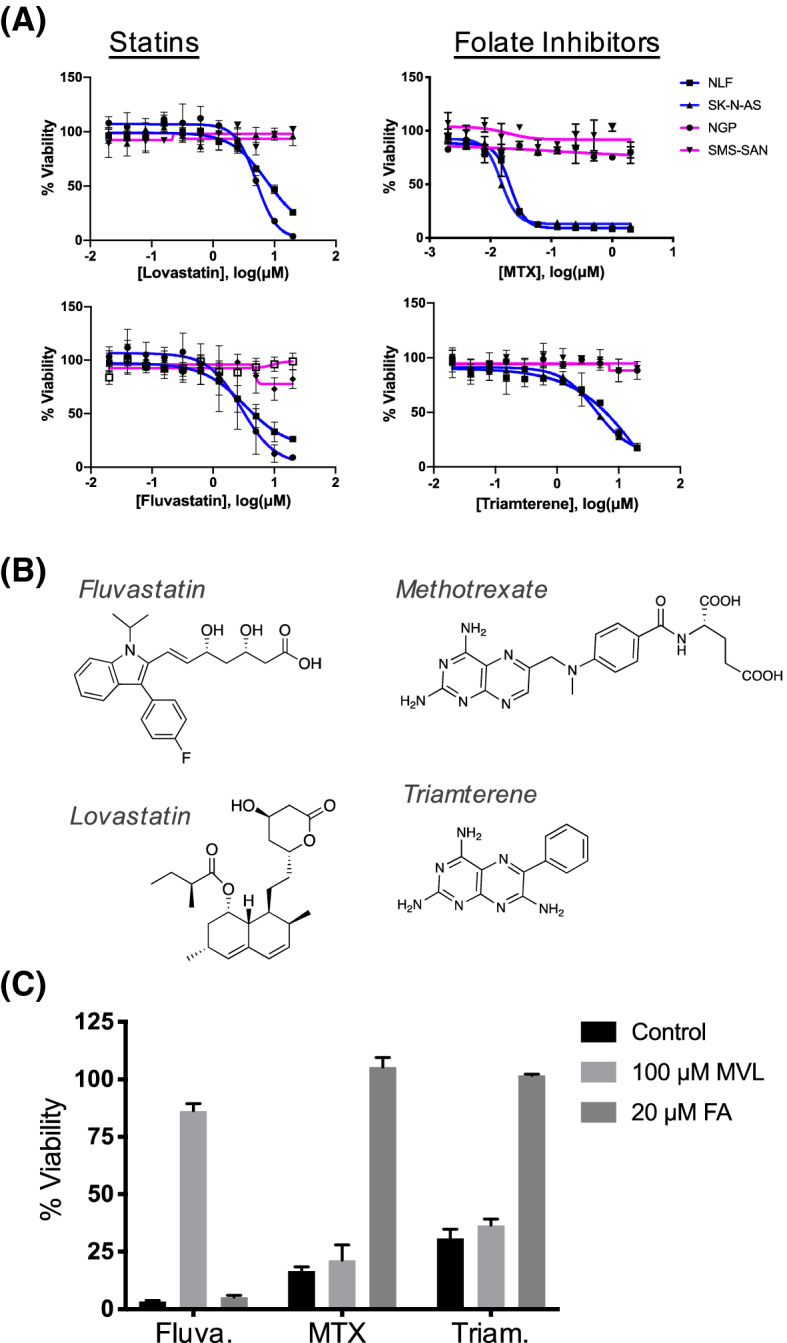
MESN subtype NBL cells are sensitive to statins and folate inhibitors. (**A**) Cell lines treated for 48 h across a range on concentrations; blue lines indicate MESN subtype, while magenta indicates MYCNA. Error bars indicate standard deviation of three biological replicates. (**B**) Chemical structures of two statins (fluvastatin; lovastatin) and two folate inhibitors (methotrexate; triamterene). (**C**) Cell viability of NLF cells treated with fluvastatin (10 µM), methotrexate (0.25 µM), or triamterene (10 µM) with or without mevalonolactone (MVL) and folic acid (FA) for 48 h. Columns indicate percent viability relative to untreated control ± standard deviation of three biological replicates.

To identify subtype-selective inhibitors, ~ 3,200 bioactive molecules from the NIH Clinical Collection and NCI Diversity Set were screened at 20 µM for 72 h to identify molecules lethal to at least one cell line. Lethal molecules were then re-screened across a concentration series ranging from 20 to 0.2 µM for 48 h. Compounds that exhibited a fourfold lower IC_50_ value in the two MESN cell lines compared to the two MYCNA controls, were selected as MESN-specific. This analysis revealed statin drugs, including fluvastatin and lovastatin, as well as the folate inhibitors methotrexate (MTX) and triamterene, as selective inhibitors of the MESN cell lines (Fig. 1A).

Six FDA-approved statins were tested across NBL cell lines to identify the most selective compound. Lovastatin, simvastatin, and fluvastatin had similar selectivity, in that MESN cells were sensitive and MYCNA cells were unaffected up to 20 µM (Supplemental Figure S1C). The hydrophilic statins pravastatin and rosuvastatin had little activity in the cell lines tested, consistent with earlier studies in neuronal development^17^. MYCN activation is often associated with drug-resistance in patients, so we tested whether MYCN expression was sufficient to drive statin resistance in NBL cells. SHEP-21N cells, which harbor a doxycycline-repressible MYCN cassette (“Dox-Off”)^18^ were treated with lovastatin for 48 h, in absence or presence of doxycycline (Supplemental Figure S1D,E). MYCN expression did not affect viability in cells treated with lovastatin, suggesting that MYCN is not sufficient to drive statin resistance.

MTX is a competitive folate analog that binds and inhibits the active site of dihydrofolate reductase (DHFR), blocking the production of dihydro- and tetrahydrofolate. These are necessary precursors to a number of metabolic pathways, including one-carbon metabolism and nucleotide biosynthesis^19^. While MTX is used as a cytotoxic chemotherapeutic compound, triamterene is generally not administered as an antifolate despite sharing structural similarities (Fig. 1B; Supplemental Figure S1B)^20^. Instead, triamterene is prescribed as a diuretic that acts by blocking renal epithelial Na^+^ transporters, and sensitive patients can be prescribed folic acid supplements to offset nutritional deficiency caused by the drug^20^.

Although statins and folate inhibitors both have well-defined mechanisms of action, we validated that these compounds target independent metabolic vulnerabilities of MESN NBL in chemical complementation experiments. Specifically, MESN NLF cells were treated with 10 µM fluvastatin in combination with either 20 µM folic acid (FA) or 100 µM of mevalonolactone (MVL). Supplementing growth media with MVL rescued cells from the inhibitory effect of fluvastatin, whereas the addition of FA had no effect (Fig. 1C). In a complementary experiment, NLF cells were treated with folate inhibitors (0.25 µM MTX or 10 µM triamterene) and supplemented with MVL or FA. Addition of FA restored viability to cells treated with MTX or triamterene, confirming that these two classes of compounds act through distinct cellular pathways.

### Statins are lethal to MESN cells by disrupting protein prenylation

**Figure 2:**
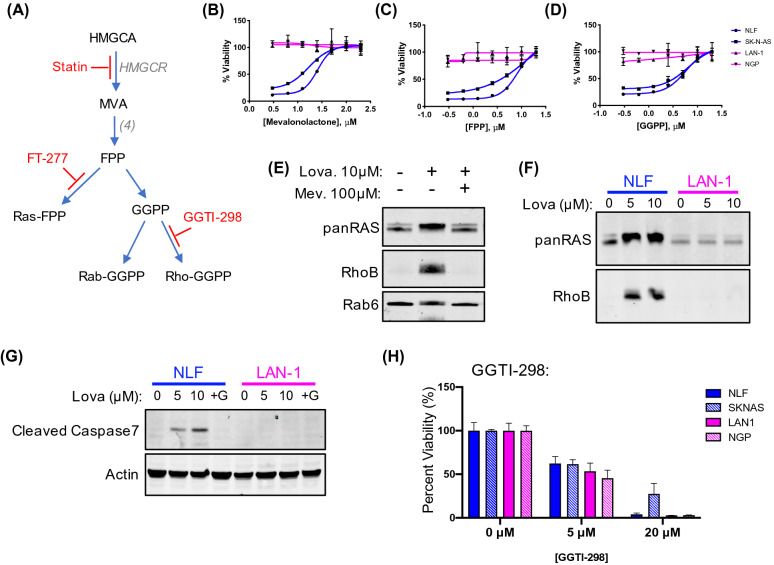
Subtype selectivity of mevalonate inhibition occurs upstream of farnesyl pyrophosphate. (**A**) Schematic drawing of isoprenoid biosynthesis and chemical inhibitors. (**B**–**D**) Four NBL cell lines treated with 20 µM lovastatin for 48 h in combination with downstream mevalonate products: mevalonolactone (MVL), farnesyl pyrophosphate (FPP), and geranylgeranyl pyrophosphate (GGPP). Blue lines indicate MESN; magenta indicates MYCNA subtype. Error bars indicate standard deviation of three biological replicates. (**E**) Western blot of RAS, RhoB and Rab6 proteins from NLF cells treated with lovastatin and MVL for 24 h. Gel images were cropped for clarity; full images available in Supplemental Figure S2. (**F**) Western blot analysis of RAS and RhoB prenylation status in NLF and LAN-1 cells treated with lovastatin for 24 h. Gel images were cropped for clarity; full images available in Supplemental Figure S2. (**G**) NLF and LAN-1 cells treated with lovastatin as indicated for 24 h. Western blot for cleaved caspase 7, and rescue by 10 µM GGPP. Gel images were cropped for clarity; full images available in Supplemental Figure S2. (**H**) Four NBL cell lines treated with GGTI-298 for 48 h; blue bars indicate MESN subtype and magenta bars indicate MYCNA subtype. Bars indicate percent viability relative to non-treated control ± standard deviation of three biological replicates.

Statins inhibit mevalonate biosynthesis, a metabolic precursor to lipids built from multiple five-carbon isoprenoid units^21^. These are appended as hydrophobic modifications to proteins, thus facilitating association with the plasma membrane^22^. Inhibition of protein prenylation is one known mechanism through which statins are lethal to cancer cell lines^8,9,13,22^. To confirm that isoprenoid depletion is lethal to NBL cells, SK-N-AS, NLF, SK-N-Be2 and IMR-32 cells were co-treated with 10 µM lovastatin and isoprenoid products of the mevalonate pathway. Co-treatment with MVL fully rescued their viability at 50 µM (Fig. 2B), confirming that lovastatin is lethal to MESN cells through mevalonate inhibition. Farnesyl pyrophosphate (FPP) and geranylgeranyl pyrophosphate (GGPP) were then tested across a range of concentrations to assess whether isoprenoids were necessary downstream metabolites. Addition of either FPP or GGPP rescued viability in MESN cells, while having no effect on MYCNA cells (Fig. 2C,D), suggesting that statins’ lethality in MESN NBL is driven by inhibition of isoprenoid biosynthesis.

The RAS family of small GTPases relies on protein prenylation to facilitate signaling at the plasma membrane^22^. As a way of evaluating the effect of lovastatin on protein prenylation, analysis of NBL cells treated with lovastatin and mevalonate was performed by western blot. Unprenylated proteins migrate slightly more slowly through a gel matrix, which can be visualized as a small shift in band localization^23–25^. In this way, changes in RAS and Rab prenylation can be observed as subtle shifts in protein gel migration^23,25^. Inhibition of RhoB prenylation results in the induction of unprenylated RhoB protein, which is dependent on de novo protein translation^26^. In some cancer lines, RhoB is typically expressed below detectable levels, but is induced by prenyltransferase inhibition and accumulates in cells^26^. These assays enabled us to evaluate whether lovastatin blocked prenylation of three GTPase classes (RAS, Rho and Rab).

To assess the effect of statins on protein prenylation, NLF cells were treated with 10 µM lovastatin in absence or presence of 100 µM MVL for 24 h, followed by western blot analysis of protein prenylation. In the control lanes, the panRAS antibody detects two bands, representing overlapping HRas and NRas bands at ~ 21 kDa and KRas at ~ 23 kDa. Lovastatin caused a shift in band migration, consistent with inhibition of RAS farnesylation (Fig. 2E,F, Supplemental Figure S2A,B). Supplementing growth media with 100 µM MVL abrogated this effect, restoring normal banding pattern. Similar shifts in gel migration were observed using a Rab6-specific antibody, in which treatment with lovastatin induced a subtle shift in Rab6 gel migration that was restored by supplementing the media with MVL (Fig. 2E, Supplemental Figure S2A). Similarly, RhoB induction upon prenylation inhibition was observed in response to lovastatin treatment, which was rescued by the addition of MVL (Fig. 2E; Supplemental Figure S2A).

We next evaluated whether differences in prenylation underpinned subtype selectivity of the compounds. Ras prenylation was assessed by gel migration in MESN NLF cells and in MYCNA LAN-1 cells, following treatment with lovastatin. Statin treatment blocked Ras prenylation in NLF, but not in LAN-1 (Fig. 2F), demonstrating subtype-specific shift in gel migration following treatment with lovastatin. Similarly, lovastatin induced apoptosis in NLF cells, and not in LAN-1, as evidenced by cleaved caspase 7 accumulation (Fig. 2G). This effect was prevented by addition of 10 µM GGPP, highlighting the connection between prenylation status and selective lethality of mevalonate inhibition. Together, these data confirm that lovastatin disrupts prenylation and induces apoptosis in MESN cells, while having little effect on MYCNA cells.

Selective prenyltransferase inhibitors were used to disrupt protein prenylation, to assess which mechanism is necessary for NBL cell viability. Ras GTPases are farnesylated by *farnesyltransferase (FT)*, whereas Rho and Rab proteins are geranylgeranylated by *geranygeranyltransferase1 (GGTase1)* and *geranygeranyltransferase2/RabGGTase (GGTase2)*, respectively. Statins block prenylation of all three GTPase classes by acting upstream in the mevalonate pathway, which provides precursors for isoprenoid biosynthesis. Selective prenyltransferase inhibitors can block prenylation of specific GTPases (Fig. 2A); FTI-277 is a selective FT inhibitor while GGTI-298 is a cell permeable selective GGTase1 inhibitor (Supplemental Figure S3A–C)^13,27^. Treatment with FTI-277 had no effect on cell viability, despite disruption of RAS farnesylation, suggesting that FT-dependent RAS farnesylation is not essential for MESN viability under the conditions tested (Supplemental Figure S3D,E). In contrast, both MESN and MYCNA cells were equally sensitive to GGTase1 inhibition by GGTI-298 (Fig. 2H), indicating that geranylgeranylation is essential for both subtypes.

### Differential *HMGCR* expression drives statin sensitivity

**Figure 3:**
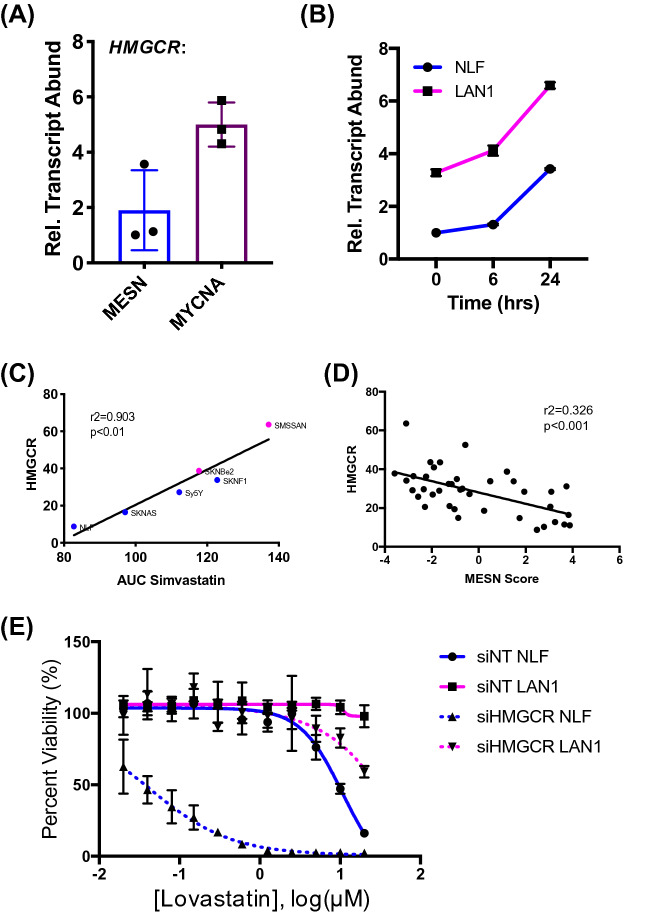
*HMGCR* expression in NBL cells drives statin sensitivity. (**A**) *HMGCR* transcript abundance in six NBL lines, measured by qPCR. Blue bars indicate average of three MESN cell lines, magenta bar indicates MYCNA lines. Error bars indicate standard deviation of three biological replicates. (**B**) NLF and LAN-1 cells treated with 10 µM lovastatin across time; *HMGCR* transcript quantified by qPCR. (**C**) *HMGCR* expression correlates with simvastatin AUC. (**D**) *HMGCR* expression negatively correlated with MESN signature across NBL cell lines. (**E**) Knockdown of *HMGCR* by siRNA, followed by treatment with lovastatin for 48 h.

Statins inhibit HMGCR, the rate-limiting step of the mevalonate biosynthetic pathway^6,28^. To assess whether changes in target abundance may drive statin sensitivity, differences in *HMGCR* transcript abundance was measured by quantitative polymerase chain reaction (RT-qPCR) in both MESN and MYCNA cell lines. Increased *HMGCR* transcript abundance was observed in statin-resistant MYCNA cell lines (Fig. 3A), suggesting differential *HMGCR* regulation between the two subtypes. As *HMGCR* expression is feedback-regulated by changes in downstream metabolic products, *HMGCR* is induced upon statin treatment^6^. To test whether the feedback mechanism that stabilizes *HMGCR* expression is dysregulated in NBL, NLF and LAN-1 cells were treated with lovastatin and *HMGCR* transcript measured across time. Both cell lines responded equally to statin treatment, confirming that the feedback mechanisms regulating *HMGCR* expression is functional (Fig. 3B). Thus, statin sensitivity is likely not caused by an impaired cellular response to treatment.

To assess the relationship between HMGCR and statin sensitivity, *HMGCR* expression was evaluated using publicly-available RNA-Seq expression profiles of 39 common NBL cell lines^29^, and compared to area under the curve (AUC) values of simvastatin sensitivity. This revealed tight correlation between statin sensitivity and *HMGCR* abundance (r^2^ = 0.903, *p* < 0.01; Fig. 3C). *HMGCR* expression was then plotted against the normalized enrichment score (NES) for the MR-signature that defines the MESN subtype, showing inverse correlation between *HMGCR* expression and MESN activity signature, with a modest but statistically significant R^2^ value (R^2^ = 0.326; *p* < 0.001; Fig. 3D). These data suggest that *HMGCR* expression is suppressed in MESN cells, and that reduced abundance may drive statin sensitivity.

To assess the functional relevance of *HMGCR* expression in statin sensitivity, *HMGCR* transcript was depleted using siRNAs. Knockdown efficiency was validated in NLF and LAN-1 cells by qPCR (Supplemental Figure S4A). After confirming inhibition of expression, the effect of *HMGCR* suppression on statin sensitivity was tested by comparing response of siHMGCR-treated cells to control cells treated with non-targeting siRNAs (siNT). Suppression of *HMGCR* conferred sensitivity to the MYCNA cell line, and induced further hypersensitivity in MESN cells, supporting the relationship between *HMGCR* expression and statin sensitivity in neuroblastoma^30,31^ (Fig. 3E). These findings were validated across statin compounds by confirming similar results with atorvastatin and cerivastatin (Supplemental Figure S4B,C).

### TGFBR2 signaling contributes to statin sensitivity in MESN NBL

**Figure 4:**
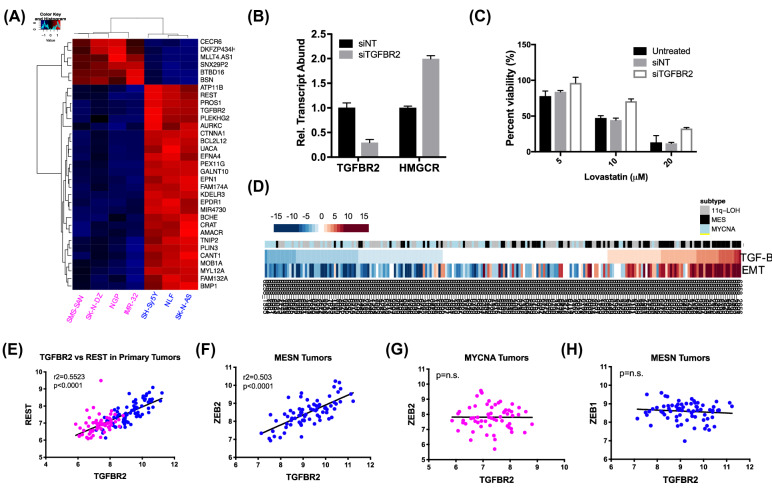
Expression analysis reveals TGFBR2 regulation of statin sensitivity through *HMGCR*. (**A**) Heatmap representing row-normalized expression values of 33 differentially expressed transcripts (*t*-test, *p* < 0.002). Image was generated using the heatmap.2 function in R^39^. (**B**) qPCR transcript analysis following treatment with siRNAs targeting *TGFBR2*; non-targeting random siRNAs used as control (siNTs). (**C**) Treatment with lovastatin following knockdown of TGFBR2 by siRNAs. (**D**) Pathway enrichment analysis of EMT and TGF-b signaling in NBL primary tumors (TARGET cohort; n = 249) revealed close association with MESN tumor subtype. Image generated using pheatmap function in R. (**E**) *TGFBR2* and *REST* expression are elevated in MESN tumors and correlated across tumors. (**F**–**G**) Linear regression analysis of *TGFBR2* and *ZEB2* expression across MESN primary tumors; no statistically significant correlation was observed in MYCNA tumors. (**H**) Linear regression analysis of *ZEB1* and *TGFBR2* expression across MESN tumors reveals no significant correlation.

Cell line expression profiles were then analyzed to identify other signaling and regulatory factors associated with statin sensitivity. By comparing profiles of four statin-resistant cell lines with three statin-sensitive lines, a cohort of 32 differentially expressed transcripts was identified (*p* < 0.002; Fig. 4A). Differentially-expressed transcripts included transforming growth factor beta receptor 2 (*TGFBR2*), a key regulator of TGF-β signaling^32^, and repressor element 1 silencing transcription factor (REST). These were notable because TGF-β signaling factors and REST were previously identified as core regulatory drivers of MESN NBL, through recent network-based analysis of NBL primary tumors^4^.

*TGFBR2* expression was then knocked down by siRNAs, to evaluate the regulatory relationship between *HMGCR* and *TGFBR2* expression in MESN cells. *TGFBR2*-specific siRNAs were transfected into NLF cells and transcript suppression was evaluated by qPCR. Treatment with siTGFBR2 resulted in 70% *TGFBR2* mRNA expression knockdown, which was sufficient to double *HMGCR* transcript abundance (Fig. 4B). Given that *HMGCR* expression modulates statin sensitivity, we hypothesized that siTGFBR2 treatment would reduce lovastatin sensitivity. To test this hypothesis, cells were treated with 10 µM and 20 µM lovastatin for 24 h following knockdown of *TGFBR2* expression. Treatment with siTGFBR2 conferred resistance to lovastatin in the sensitive NLF line, supporting the hypothesis that TGF-βR2 signaling promotes statin sensitivity in MESN NBL (Fig. 4C).

Gene set enrichment analysis of transcripts associated with epithelial-to-mesenchymal transition (EMT) and TGF-β signaling was used to evaluate the role that these processes play in MESN NBL. By ranking tumor expression profiles by TGF-β enrichment, a close association between EMT and TGF-β was observed across primary tumors (Fig. 4D), highlighted a well-established link between EMT and TGF-β signaling^33,34^. The expression of TGF-β signaling effectors was then assessed from NBL primary tumor profiles. Comparing MYCNA and MESN profiles revealed that, similar to cell lines, both *TGFBR2* and *REST* expression were elevated in MESN tumors and tightly correlated across samples, as determined by linear regression analysis (r^2^ = 0.5523; *p* < 0.0001; Fig. 4E; Supplemental Figure S5A,B).

Previous studies in breast cancer found that ZEB1-dependent EMT conferred sensitivity to fluvastatin-induced apoptosis^10^. To test whether a similar mechanism may drive sensitivity in MESN NBL, the relationship between *TGFBR2* expression and transcripts of two EMT effectors, ZEB1 and ZEB2, was compared across tumor profiles by linear regression analysis. *TGFBR2* and *ZEB2* transcripts were significantly correlated across MESN primary tumors, yet no such correlation could be observed in MYCNA or stage 1 primary tumors (Fig. 4F,G; Supplemental Figure S5C). No correlation was observed between *TGFBR2* and *ZEB1* across tumors (Fig. 4H; Supplemental Figure S5D,E), suggesting that *TGFBR2-ZEB2* signaling is preferentially active in the MESN NBL subtype.

## Discussion

By screening for subtype-selective molecules, tractable metabolic dependencies were revealed that underpin the MESN NBL subtype. These biosynthetic pathways are essential to support cell viability, and represent vulnerabilities that may be exploited therapeutically. Statin sensitivity may be derived from regulatory changes adopted during MESN tumor development. Among the 25 putative Master Regulators driving MESN NBL were core TGF-β effectors that support MESN tumor development^4^. Induction of TGF-β signaling pathway, and specifically *TGFBR2,* appears in part to regulate statin sensitivity in NBL by suppression of the drug target HMGCR, creating an “Achilles’ heel” that might be exploited to induce selective apoptosis.

Statin selectivity has been reported across a variety of cancer cell types, and lovastatin induces apoptosis through inhibition of geranylgeranylation in acute myeloid leukemia and multiple myeloma^9,13,31^. Although many responses to statins are shared cross cell types, defining global features of sensitivity has been challenging. For example, a ten-gene fluvastatin sensitivity signature in breast cancer cells shares little overlap with either the MESN gene expression signature or the differentially expressed transcripts identified in resistant NBL lines^8^, suggesting that cancer-specific mechanisms likely underpin cellular responses. The MESN gene signature served as a predictive marker of statin sensitivity in NBL, and it would be interesting to evaluate its predictive power across cancers. MESN cell markers, such as vimentin, have been associated with statin-sensitive cell lines^10,11^, suggesting that the MESN gene signature could have utility as a predictor of sensitivity in adult cancers as well.

Some epidemiological studies have suggested that statins do not significantly reduce the cancer risk, however these studies often do not take into account tumor subtype stratification or other molecular features that define statin sensitivity^35, 36^. In contrast, classification of breast cancer patients based on tumor subtype found that fluvastatin treatments reduced proliferation rates and induced apoptosis in high-grade breast cancers^37^, and that patients taking lipophilic statins were less likely to develop estrogen receptor-negative (ER-) breast cancer^38^. A greater understanding of factors influencing statin sensitivity would enable clinicians to draw meaningful comparisons and accurately assess whether statins have an impact on tumor development. It is proposed that the MESN expression signature could be used to define a sensitive patient population that would respond to mevalonate inhibition in the clinic.

## Materials and methods

### Cell culture and chemical treatments

Cell lines were acquired from the American Type Culture Collection (ATCC), and grown in medium composed of Advanced RPMI growth media (Gibco), 10% fetal bovine serum (FBS), 1% Penicillin/Streptomycin, and 1% GlutaMax glutamine supplement (Gibco). To generate dose–response series, cells were trypsinized and reseeded in 384w plates at a density of 1,000 cells/well using a BioMEK liquid handling robot under sterile conditions (Beckman Coulter). The following day, chemicals were diluted to create appropriate concentrations in assay plates. Plates were incubated at 37 °C and 5% CO_2_ for 48 h. Cell viability was quantified by measuring bioluminescence following the addition Cell Titer Glo (Promega) to assay plates, following manufacturer’s instructions.

### Protein detection by western blot

Cells were seeded in 6 w plates at a density of 400k cells/well and incubated overnight. The following day, compounds were diluted from 10 mM DMSO stocks to create treatment groups, with DMSO was added to control wells to maintain equal concentration across groups. After 24 h treatment, cells were trypsinized and pelleted in eppendorf tubes. Pellets were incubated on ice in RIPA cell lysis buffer, followed by centrifugation at 17,000×*g* for 10 min. Protein was denatured by boiling in 1× laemmli buffer, and separated by gel electrophoresis using NuPAGE 4–12% Bis-Tris protein gels (Thermo Fisher Scientific). Semi-dry protein transfer to nitrocellulose membrane was performed using iBlot 2 dry transfer system (Thermo Fisher Scientific), following manufacturer’s instructions. Membranes were visualized using LICOR Odyseey imaging system following manufacturer’s instructions (LICOR Biosystems). All antibodies were purchased from Cell Signaling Technologies, and used at 1:1,000 dilutions in blocking buffer.

### siRNA treatment and gene expression analysis

Pooled small interfering RNAs (siRNAs) targeting *HMGCR* or *TGFBR2* (SmartPOOL; Dharmacon) were transfected into NLF and LAN-1 cells using lipofectamine RNAi/MAX transfection reagent (Thermo Fisher Scientific), following a protocol modified from manufacturer’s instructions. Two transfections were performed to achieve satisfactory knockdown of transcript abundance. Reverse transfection of cells was achieved by adding 300 µL of siRNA-lipid complexes to wells of a 6 w plate, and incubating at 37 °C for 10 min. Cells were then added to the complexes for a final density of 250,000 cells/well. The following day, the media was removed and fresh media was added and incubated for 2 h at 37 °C. For a second round of forward transfection, fresh siRNA-lipid complexes were created in 300 µL of Opti-mem media (Gibco). Complexes were then added drop-wise to wells containing cells and allowed to incubate for another 24 h. Following this 24 h period, cells were either sampled for qPCR analysis of gene expression, or reseeded into 384w plates for lovastatin treatment.

Reverse transcription quantitative polymerase chain reaction (RT-qPCR) analysis was used to quantify transcript abundance from target genes of interest. Total RNA was isolated from cells using the RNeasy RNA isolation kit (QIAGEN), following manufacturer’s instructions. Total RNA abundance and purity was determined using a nanodrop spectrophotometer (Thermo Fisher Scientific). Two micro grams of total RNA was used as a template to generate cDNA using both oligo-dT and random priming hexamers. cDNA was treated with RNAse A to remove residual RNA, and diluted tenfold for qPCR reactions using SYBR green (Invitrogen) and gene-specific qPCR primers (Supplemental Table T1). qPCR reactions were performed using the Viia7 Real-Time PCR system (Applied Biosystems), and relative transcript abundance evaluated using the deltaCT method, with GAPDH housekeeping gene as normalization control.

Transcriptome analysis was performed using publicly-available RNA-Seq gene expression profiles^29^. Profiles from NBL cell lines were downloaded from gene expression omnibus (GEO; Accession GSE89413) Differential expression analysis was performed using the Bioconductor suite in R statistical programming language^39^ (R Development Core Team; www.R-project.org.

Gene expression data from the TARGET cohort (n = 249) were collected as described^4^. Gene expression signatures were generated as z-scores as follows. For each sample, genes were ranked based on their expression. Next, for each gene, its median across samples was subtracted to the expression value, and then divided by the median absolute deviation. Pathway analysis was performed on the resulting transformed matrix using the Hallmarks of Cancer gene set from the Broad Institute (https://www.gsea-msigdb.org/gsea/msigdb/genesets.jsp?collection=H). Enrichment analysis was performed for the pathways TGF_BETA_SIGNALING and EPITHELIAL_MESENCHYMAL_TRANSITION using an analytical rank-based enrichment analysis (see aREA function from the *VIPER* package version 1.19.3). To show correlation between the selected pathways and subtyping, we sorted the samples based on the TGF_BETA_SIGNALING pathway. An heatmap showing the clustering based on these 2 pathways was generated using *pheatmap* version 1.0.12. Annotation about three NBL subtypes was used as described^4^. All analyses were performed using R version 3.6.1. All packages used for the analysis are available through Bioconductor for R.

## Supplementary information

Supplementary information
