# Supplementary material for: Mesenchymal subtype neuroblastomas are addicted to TGF-βR2/HMGCR-driven protein geranylgeranylation
Source: Sci Rep. 2020 Jul 1;10:10748. doi: 10.1038/s41598-020-67310-0 (PMC7329873; doi:10.1038/s41598-020-67310-0)
Supplement: Supplementary file 1 — Supplementary information [file 41598_2020_67310_MOESM1_ESM.pdf]

SUPPLEMENTAL INFORMATION

Mesenchymal subtype neuroblastomas are addicted to TGF-βR2/ HMGCR-driven protein geranylgeranylation

Michael E. Stokes[1], Jonnell Candice Small[1,2], Alessandro Vasciaveo[3], Kenichi Shimada[1,4], Tal Hirschhorn[1], Andrea Califano[3], Brent R. Stockwell*[1,6]

[1]Department of Biological Sciences, Columbia University, New York City, NY 10027, USA

[2]Current Address: Department of Medicine, Harvard Medical School, Boston, MA 02115, USA. Chemical Biology and Therapeutic Sciences Program, Broad Institute, Cambridge, MA 02142, USA

[3]Department of Systems Biology, Columbia University, New York City, NY 10027, USA

[4]Current address: Laboratory of Systems Pharmacology, Harvard Medical School, Boston, MA 02115, USA

[6]Department of Chemistry, Columbia University, New York City, NY 10027, USA

CORRESPONDING AUTHOR

Brent R. Stockwell

Department of Biological Sciences and Department of Chemistry

Columbia University

1208 Northwest Corner Building, MC4846, 550 West 120th St

New York City, NY, USA

bstockwell@columbia.edu

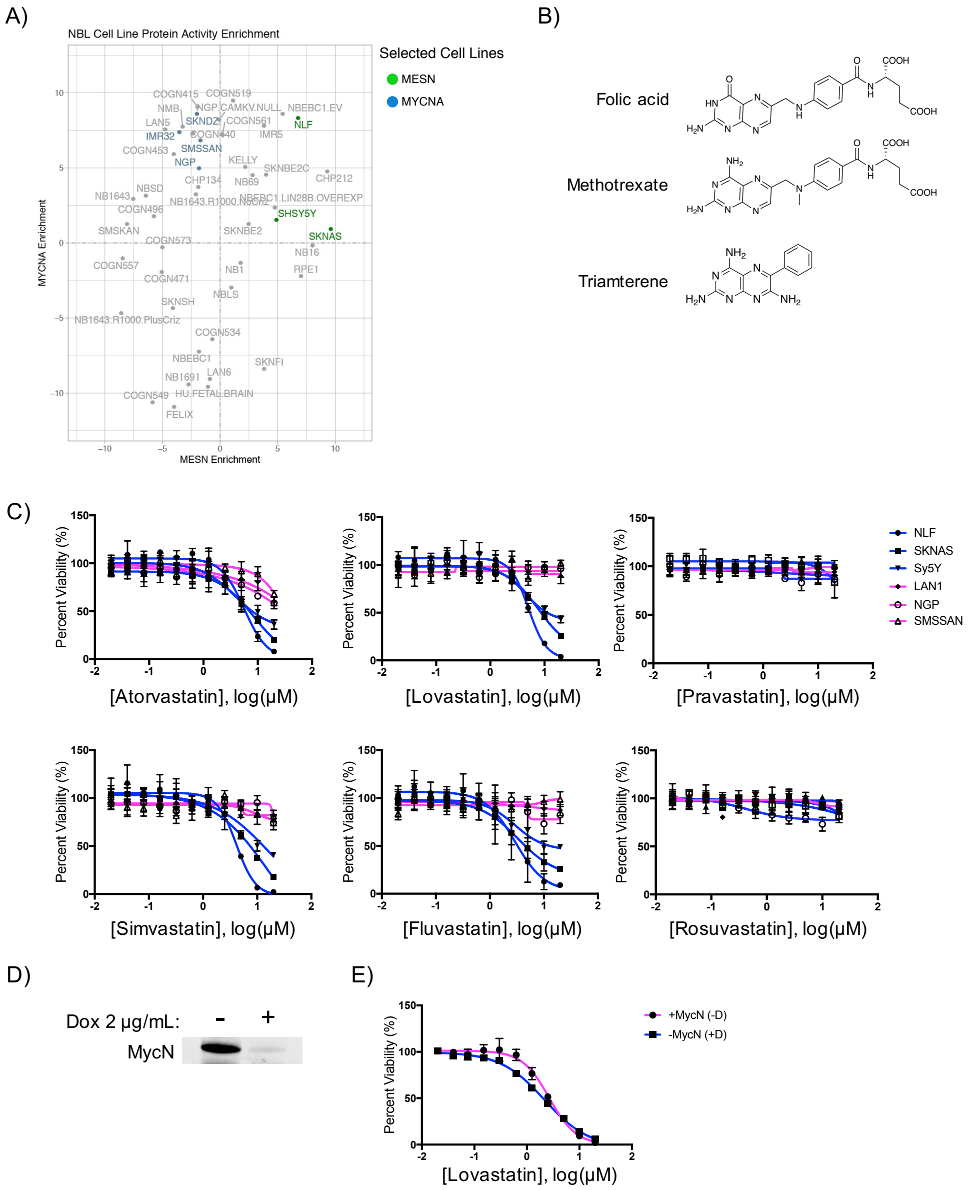

Supplemental Figure S1. (A) Scatter plot of cell line transcriptional profiles arranged by enrichment for the MYCNA and MESN master regulator (MR) proteins. Highlighted in green are the MESN cell lines used in the present study; blue font indicates MYCNA cell models. (B) Chemical structures of folic acid and the folate inhibitors methotrexate and triamterene. (C) Six NBL cell lines treated to six FDA-approved statins. Cells treated for 48 h across series of concentrations; blue lines indicate MESN cell models, magenta indicates MYCNA cell lines. Error bars indicate standard deviation of three biological

replicates. (D-E) MycN-inducible expression in MESN cells by “Dox-off” expression cassette. SHEP-21N cells treated with lovastatin for 48 h in absence or presence of 2 µg/mL doxycycline. Error bars indicate standard deviation of three biological replicates.

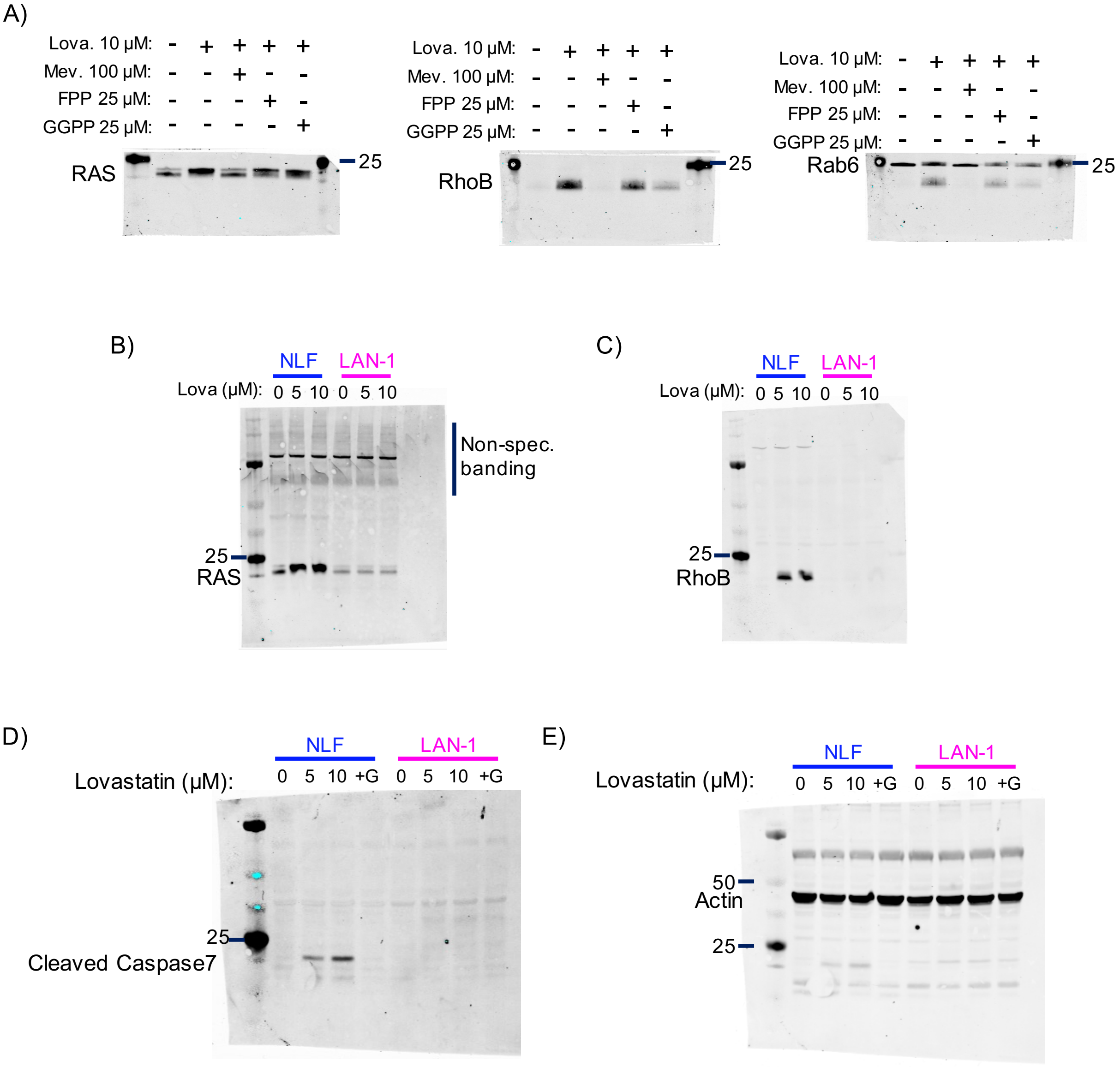

Supplemental Figure S2. (A) Western blot analysis panRAS, RhoB and Rab6 prenylation in NLF cells following 24 h treatment with 10 µM lovastatin in combination with mevalonolactone (100 µM), farnesyl pyrophosphate (FPP; 25 µM), or

geranylgeranyl pyrophosphate (GGPP; 25 μM). Membranes were cut prior to focus staining on specific region of interest. (B) RAS prenylation in NLF (MESN subtype) and LAN-1 cells (MYCNA subtype) following treatment with 10 μM lovastatin for 24 h. (C) RhoB expression in NLF (MESN subtype) and LAN-1 cells (MYCNA subtype) following treatment with lovastatin for 24 h. (D-E) Cleaved caspase7 abundance in NLF (MESN) and LAN-1 (MYCNA) cell lines treated with lovastatin for 24 h. NLF and LAN-1 cells rescued by supplementing growth media with 10 μM GGPP (+G). Membranes were stripped and reblotted with an actin-specific primary antibody to ensure equal loading across samples (E).

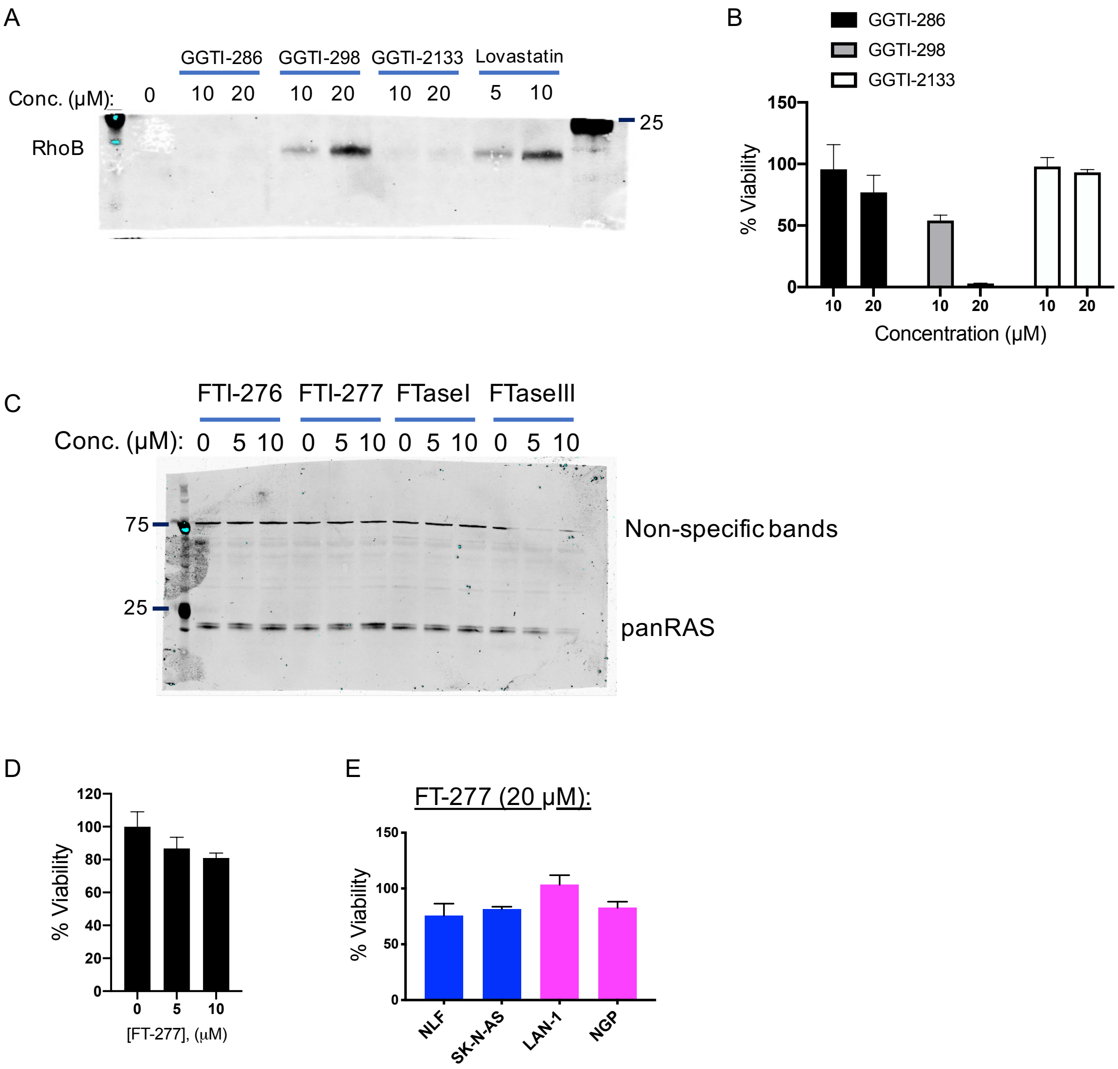

Supplemental Figure S3. (A) Western blot analysis of RhoB accumulation in NLF cells following 24 h treatment with putative geranylgeranyltransferase inhibitors (GGTIs). (B) NLF cell viability following treatment with GGTIs for 48 h. Columns indicate percent viability relative to untreated control, mean ± standard deviation. (C) Western blot analysis of RAS prenylation status in NLF cells following 24 h treatment with FTI-277. (D) NLF cell viability following treatment with farnesyltransferase inhibitor FTI-277 for 48 h. Columns indicate percent viability relative to untreated control, mean ± standard deviation. (E) Four NBL cell lines treated with 20 μM FTI-277 for 48 h. Blue bars indicate MESN subtype;

magenta bars indicate MYCNA subtype. Values represent mean of three biological replicates ± standard deviation.

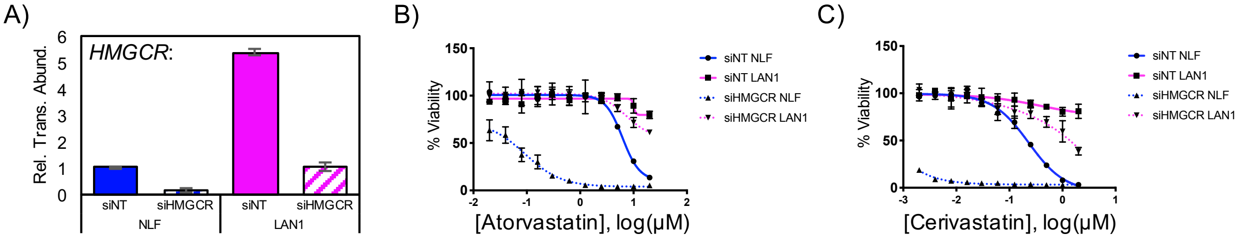

Supplemental Figure S4. (A) q-PCR quantification of *HMGCR* transcript of NLF and LAN-1 cell lines following treatment with *HMGCR*-specific siRNAs. Columns indicate transcript abundance relative to NLF cells treated with non-targeting siRNAs; mean of three biological replicates ± standard deviation. (B) siHMGCR knockdown cell lines treated with atorvastatin for 48 h. (C) siHMGCR knockdown cell lines treated with cerivastatin for 48 h. Error bars indicate standard deviation of three biological replicates.

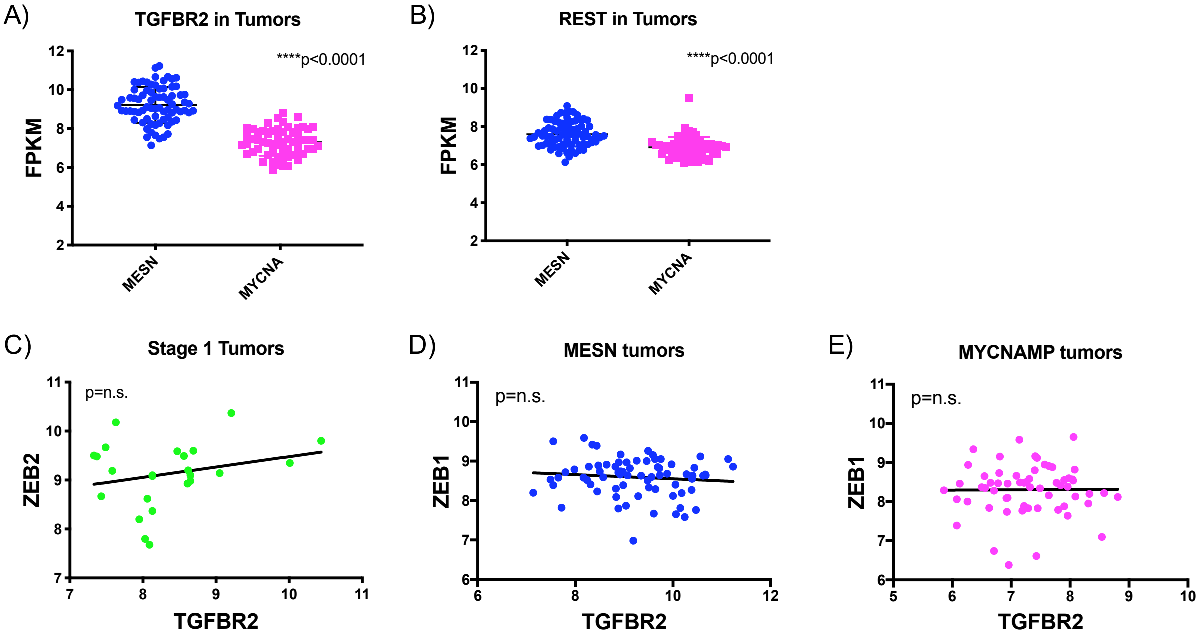

Supplemental Figure S5. (A-B) TGFBR2 and REST transcript abundance in MESN and MYCNA NBL primary tumors. (C) Linear regression analysis of ZEB2 and TGFBR2 in Stage 1 NBL primary tumors. (D) Linear regression analysis of ZEB1 and TGFBR2 in MESN NBL primary tumors. (E) Linear regression analysis of ZEB1 and TGFBR2 in MYCNA NBL primary tumors.

qPCR Primer Name	Sequence
GAPDH FW	*5' CTCCAAAATCAAGTGGGGCG 3'*
GAPDH RV	*5' ATGACGAACATGGGGGCATC 3'*
TGFBR2 FW	*5' ACGCCAAGGGCAACCTACAG 3'*
TGFBR2 RV	*5' CGCAGGGAAAGCCCAAAGTC 3'*
HMGCR FW	*5' TGCAGAGCAATAGGTCTTGGTG 3'*
HMGCR RV	*5' TCGAGCCAGGCTTTCACTTC 3'*

Supplemental Table T1. Primers used in qPCR experiments.
